# Investigation of Pristine Graphite Oxide as Room-Temperature Chemiresistive Ammonia Gas Sensing Material

**DOI:** 10.3390/s17020320

**Published:** 2017-02-09

**Authors:** Alexander G. Bannov, Jan Prášek, Ondřej Jašek, Lenka Zajíčková

**Affiliations:** 1 Department of Chemistry and Chemical Technology, Novosibirsk State Technical University, K. Marx 20, RU-630073 Novosibirsk, Russia; bannov_a@mail.ru; 2 SIX Research Centre, Brno University of Technology, Technická 10, CZ-61600 Brno, Czech Republic; 3 Department of Physical Electronics, Faculty of Science, Masaryk University, Kotlářská 2, CZ-61137 Brno, Czech Republic; jasek@physics.muni.cz (O.J.); lenkaz@physics.muni.cz (L.Z.); 4 RG Plasma Technologies, CEITEC—Central European Institute of Technology, Masaryk University, Purkyňova 123, CZ-61200 Brno, Czech Republic

**Keywords:** graphite oxide, chemiresistive gas sensor, sensitivity, ammonia

## Abstract

Graphite oxide has been investigated as a possible room-temperature chemiresistive sensor of ammonia in a gas phase. Graphite oxide was synthesized from high purity graphite using the modified Hummers method. The graphite oxide sample was investigated using scanning electron microscopy, energy dispersive X-ray spectroscopy, X-ray diffraction, thermogravimetry and differential scanning calorimetry. Sensing properties were tested in a wide range of ammonia concentrations in air (10–1000 ppm) and under different relative humidity levels (3%–65%). It was concluded that the graphite oxide–based sensor possessed a good response to NH_3_ in dry synthetic air (ΔR/R_0_ ranged from 2.5% to 7.4% for concentrations of 100–500 ppm and 3% relative humidity) with negligible cross-sensitivity towards H_2_ and CH_4_. It was determined that the sensor recovery rate was improved with ammonia concentration growth. Increasing the ambient relative humidity led to an increase of the sensor response. The highest response of 22.2% for 100 ppm of ammonia was achieved at a 65% relative humidity level.

## 1. Introduction

Ammonia is noxious, irritating and, in higher concentrations, a dangerous gas. It is frequently used in the chemical industry, in oil refining and in refrigeration technology, and its detection is of great significance due to its high toxicity [1,2]. Therefore, the important issue is to create a room-temperature–operated ammonia gas sensor with enhanced characteristics of: sensor response, recovery time and response time. Usually, commercial ammonia gas sensors are based on metal oxide semiconductors; however, these sensors operate at relatively high temperatures (200–350 °C) [3,4]. Therefore, the high power consumption of these sensors is a problem to be solved by using novel active materials, one of which can be graphite oxide.

Graphite oxide (GO) is a special type of graphite-like three-dimensional material intercalated by various oxygen-containing functional groups (carboxylic groups, epoxy groups, ether groups, etc.) [5]. Also, GO may contain the water intercalated between graphene layers [6]. It was first synthesized by Brodie, in 1855, who treated the natural graphite with a mixture of potassium chlorate and fuming nitric acid [7], and then it became very intensively studied as a precursor for a set of graphene-like materials, such as graphene oxide, reduced graphene oxide, graphite nanoplatelets and graphene, in recent years. Besides the Brodie method, there are Staudenmaier [8] and Hummers [9] methods, which are also frequently used for GO synthesis. Nowadays, the Hummers method is often used to obtain GO because it is simple and less dangerous, which makes it possible to create many modifications [10,11,12], allowing the synthesis of materials with a wide range of C:O ratios and defectiveness. Pristine GO is an electrical insulator, and can become a conductor after reduction. Such a material is then called reduced graphite oxide. All materials derived from GO are much poorer in crystallinity and carrier mobility.

GO is one of the materials that can be utilized for ammonia detection. The excellent ammonia adsorption ability of graphite oxide in water was confirmed in [12,13]. Most of the articles are devoted to the creation of sensors based on graphene-like materials, such as graphene, graphene oxide and reduced graphene oxide [14,15,16]. Zhang et al. [17] created sensors based on SnO_2_ and CuO nanoflower–decorated graphene possessed the highest response of 4.9% to 300 ppm of NH_3_. In [18] the authors investigated hybrid tetralayered polyaniline (PANI)/graphene oxide/PANI/ZnO sensors for ammonia detection at the relative humidity of 65%. In [19] the authors created Cu-benzene trycarboxylic metal organic framework (MOF)/graphene-based hybrid materials showing the response of 3.7% to 500 ppm of NH_3_. Katkov et al. [20] developed fluorine-functionalized ammonia gas sensors possessing a 10% response to 10,000 ppm of NH_3_.

Graphite oxide can be successfully used for room-temperature detection of ammonia because of its good adsorption ability and the possibility to enhance the texture characteristics by its reduction [21,22,23]. However, the data on its use in an initial non-reduced state of graphite oxide as an active material for ammonia gas sensors are poorly presented and the research is mainly concentrated on graphene oxide sheets or reduced graphene oxide [24,25]. It is worth noting that graphite oxide possesses higher scalability potential in comparison with graphene oxide, which needs a long and energy-consuming stage of sonication. In addition, graphite oxide possesses the highest concentration of oxygen-containing functional groups compared with its above-mentioned derivatives, which gives a potential for GO to be used as an active material for the detection of toxic gases [12].

This work is devoted to the investigation of the ammonia gas–sensing properties of GO when used as an active layer of a chemiresistive gas sensor. The role of relative humidity on the sensor response is shown. Cross-sensitivity tests of the GO sensor in hydrogen and methane were carried out.

## 2. Experimental 

Graphene oxide used as active material for ammonia gas sensor was synthesized by modified Hummers technique. The GO synthesis technique was described in detail in [26]. Shortly, high purity artificial graphite was sieved using sieve with a mesh size of 100 µm. Then, 20 g of graphite was placed into the flask with 460 mL of concentrated H_2_SO_4_ (96%) and 10 g of NaNO_3_. The resulting mixture was mixed by the magnetic stirrer for 10 min and kept at the temperature of 0 °C in ice bath. After 15 min of synthesis beginning anhydrous KMnO_4_ (60 g) was added to the mixture of graphite/H_2_SO_4_/NaNO_3_ and the resulting mixture was kept for 20 min at 0 °C followed by the heating to 35 °C for 30 min. To perform the hydrolysis of graphite intercalated compounds, the mixture was poured into the flask with 230 mL of ice and kept at the room temperature (25 ± 2 °C) for 15 min. The last stage of synthesis was the addition of 840 mL of H_2_O_2_ and the mixture was kept for additional 15 min at the room temperature. The prepared GO was washed by deionized water and dried in air at 90 °C during 24 h. Adding the excess of hydrogen peroxide was used to make the oxidation of graphite deeper and to intensify the evolution of increased amount of oxygen.

GO was investigated by scanning electron microscopy (SEM) using Hitachi S-3400N equipped with energy dispersive spectroscopy (EDX) add-on. X-ray diffraction (XRD) of GO sample was carried out using DRON-3 spectrometer (Cu Kα, λ = 1.54 Å). Thermal behavior of the sample was investigated by thermogravimetry (TG) and differential scanning calorimetry (DSC) using NETZSCH STA 449C analyzer. Raman spectroscopy was carried out using Renishaw InVia spectrometer in the range of 100–3200 cm^−1^ (λ = 514 nm).

GO based chemiresistive gas sensor was obtained by spray coating of GO suspension over the preheated substrate at 80 °C using Fengda BD-208 airbrush. The GO suspension was prepared from 100 mg of GO powder thoroughly dispersed in 10 mL of *N*,*N*-dimethylformamide by 1 h ultrasonication in water bath and spray-coated between two 200 nm thick gold electrodes previously sputtered on a Si/SiO_2_ (535 µm/90 nm) substrate to form an active area of 2 × 4 mm. The thickness of resulted sensing layer was measured using mechanical profilometer DektaXT (Bruker, Billerica, MA, USA, DE) to be ~2 µm.

**Figure 1:**
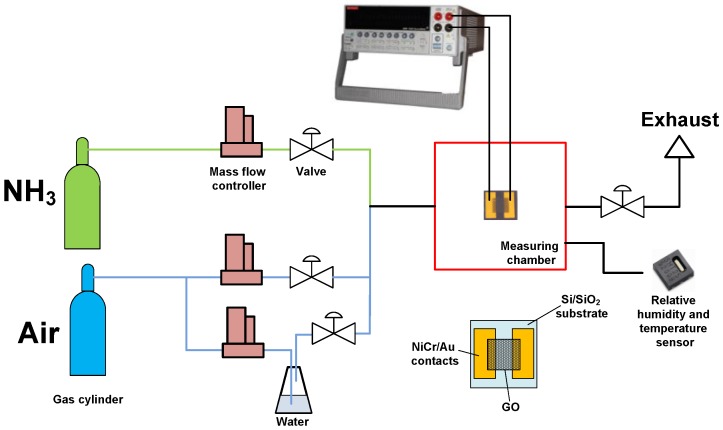
Scheme of gas rig for chemiresistive GO-based ammonia gas sensor characterization.

The response of gas sensors was examined using the custom-made gas rig (see Figure 1). The gas rig consisted of three gas channels. Synthetic air (80% N_2_ + 20% O_2_, Linde Gas, Brno, Czech Republic) was used as gas carrier. The second line was used for analyte: mixture of 5000 ppm NH_3_ in synthetic air (Linde Gas, Czech Republic). The third line was used for admixing of wet air for measurements at different relative humidity (RH) evaporated from deionized water. The main parameter of the sensors was sensor response (%):

ΔR/R_0_ = (R − R_0_)/R_0_, (1)

where R is the resistance of the sensor exposed to NH_3_, (Ω); R_0_ is the sensor resistance in synthetic air, (Ω). The investigation of sensor response was carried out at room temperature (25 ± 2 °C). The sensors were examined in a concentration range of 100–1000 ppm. The deviation of sensor response was ±0.2%.

To estimate the sensor selectivity, the sensor response measurements in admixtures of H_2_ and CH_4_ (5000 ppm of analyte diluted in air, Linde Gas, Brno, Czech Republic) were also carried out. Sensor resistance was measured by two-point technique using Keithley 2410 SourceMeter (Tektronix, Beaverton, OR, USA) at bias voltage of 1 V. Current-voltage curves of the sensor were measured by Keithley 4200-SCS Semiconductor characterization system (Tektronix, Beaverton, OR, USA) at room temperature (25 ± 2 °C).

The testing of gas sensors was carried out at different RH. RH and the temperature in the measuring chamber was monitored by calibrated SHT25 (Sensirion, Staefa, Switzerland) sensor which was placed inside the measuring chamber. The volume of the chamber where the sensors were examined was 125 cm^3^ (length −8 cm, width −6 cm, height −2.6 cm). The RH level of dry gases (air, NH_3_, H_2_, CH_4_) was on the level of 2.5%–3% during the measurement. The measurements with different RH were carried out by wet air, obtained by the bubbling of dry air, fed through the deionized water taken from Millipore (Merck Millipore, Billerica, MA, USA), and its mixing with dry air and analyte.

## 3. Results

### 3.1. GO Characterization

**Figure 2:**
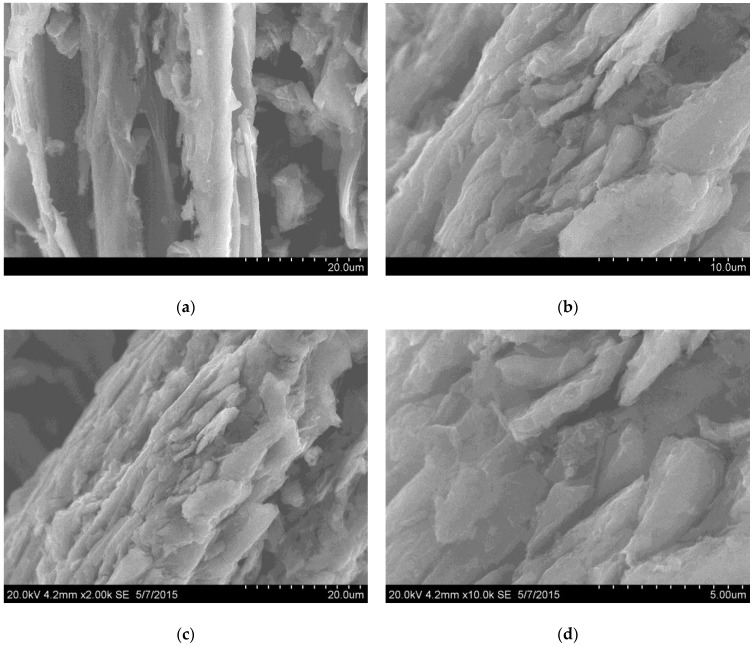
SEM images of GO sample at different magnifications (**a**)—x2000; (**b**)—x5000; (**c**)—x2000; (**d**)—x10,000). Particles of micron size are covered by platelets of GO due to strong acidic treatment.

The GO sample was successfully synthesized using the modified Hummers technique described above. SEM images of the synthesized GO sample are shown in Figure 2. The GO sample is represented by micron-sized rough particles. The particle size of the obtained material was determined by the initial size of the graphite used for the synthesis, which was separated using a sieve with a mesh size of 100 µm. From the SEM images shown in Figure 2, it is clear that the strong treatment of graphite made the material more defective and the particles’ surfaces were covered by platelets of GO. According to the EDX data, there were three main elements present in prepared GO: C (70.44 at. %), O (22.46 at. %) and S (7.1 at. %). The C:O ratio value of 3:1 confirmed the high content of oxygen-containing functional groups and it is in agreement with the previously published data corresponding to GO synthesized by the Hummers technique [6]. The content of sulfur was high enough in comparison with the data published in [6,12], and this value usually reaches 1–3 wt %; in some cases, the complete absence of sulfur was detected. The appearance of sulfur can be linked with the presence of S = O and S-O groups on the GO surface [12]. It can be suggested that sulfur can take part in the reactive adsorption of ammonia, because this effect was found in [6].

**Figure 3:**
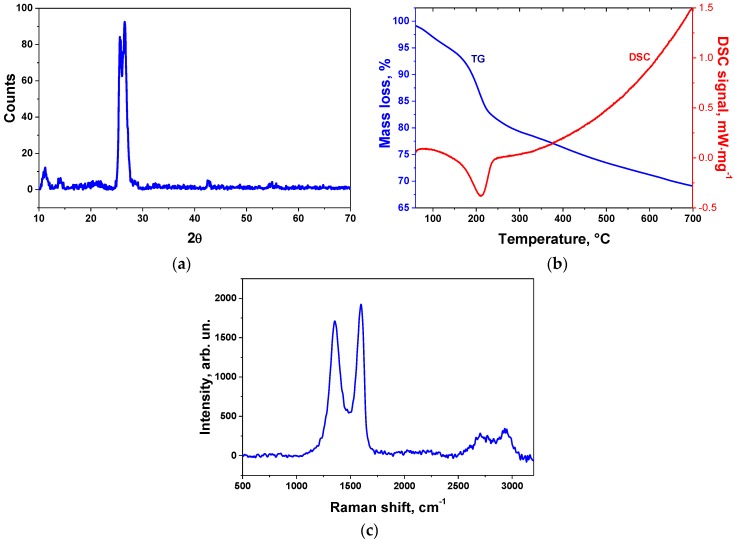
XRD pattern (**a**), TG/DSC curves (**b**), and Raman spectrum (**c**) of synthesized GO sample.

The XRD pattern of the GO sample is shown in Figure 3a. According to the XRD data, the prepared sample represents a partially oxidized GO due to the presence of the graphite phase around 2θ = 26°. There is a peak corresponding to the GO phase at 2θ = 11.3° with an interlayer distance of 7.87 Å. The presence of a peak at 2θ = 25.6° can be assigned to graphite-intercalated compounds. The DSC curves show the thermal reduction of GO with the onset of an exothermal peak of 165 °C and the end at 230 °C (the reduction enthalpy was −244 J/g). Generally, the reduction enthalpy varied from −78 to −652 J/g [27]. The value of the enthalpy of the GO reduction process was relatively low [28]. This value confirms the partial presence of a graphite phase in the active material that is linked with its higher conductivity in comparison with strongly oxidized graphites. It is worth noting that graphite oxide resistance tends to grow with the increase of the oxygen-containing functional groups concentration and it becomes an insulator in a deep oxidation state. GO reduction was accompanied by a strong mass loss of 10% in the temperature range of 165−230 °C. The beginning of GO reduction limits the sensor operation temperature which was around 100–120 °C, and exceeding it will lead to the decrease of the concentration of oxygen-containing groups which is linked with the response.

Raman spectroscopy (Figure 3c) showed the presense of two strong peaks, D (1359 cm^−1^) and G (1593 cm^−1^), corresponding to the disorder and graphitic structure of the graphite material [29]. The ratio I(D)/I(G) was equal to 0.86 which confirms the high defectiveness of the material. The ratio value is comparible with the value of highly oxidized graphite [30,31].

### 3.2. GO-Based Sensor Testing

**Figure 4:**
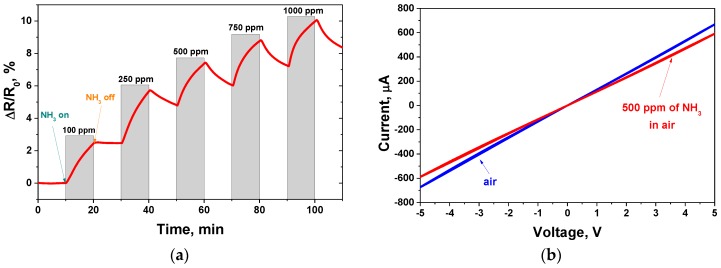
GO-based chemiresistive sensor measurements at room temperature: (**a**) response to ammonia in the range of concentrations from 100 ppm to 1000 ppm; (**b**) current-voltage characteristic measured in air atmosphere (red curve) and in 500 ppm of ammonia in air mixture (blue curve).

Successfully prepared sensors were tested for their initial resistance which was measured as 7.2 kΩ and increased during ammonia adsorption. The GO sensor response to ammonia varied from 2.5 to 10% in a concentration range from 100 to 1000 ppm and at a low RH of 3% (Figure 4a). The sensor showed a relatively high response in comparison with carbon nanotubes and graphene-like materials [32]. For example, in [33] the author obtained PANI/multi-wall carbon nanotube (MWCNT) sensors and the response of the plasma-treated PANI/MWCNT sensor was 1.6% at 100 ppm NH_3_. The obtained GO sensor showed a slightly higher response in a comparison with a reduced graphite oxide ammonia sensor (5.5% at 200 ppm) [34]. Also, the plasma-treated sensors MWCNT/Nafion with a response of 2.4% (100 ppm NH_3_) were obtained in this article. The sensor exhibited a weak recovery rate at relatively low concentrations of ammonia (e.g., 100 ppm). The increase of the NH_3_ concentration induced an increase of the recovery rate. The same effect was observed in [20], although there was no explanation given for it. It is likely that low concentrations of ammonia lead to the formation of a thin layer of molecules adsorbed on the GO surface, the biggest part of which is chemically adsorbed, while concentrations higher than ~100–250 ppm produce an increase of physically adsorbed molecules which are easy to remove from the surface.

The current-voltage curves of the sample are shown in Figure 4b. The sensor exhibited a linear I-V characteristic. The addition of NH_3_ to the air resulted in a slight change of the curve slope. 

**Figure 5:**
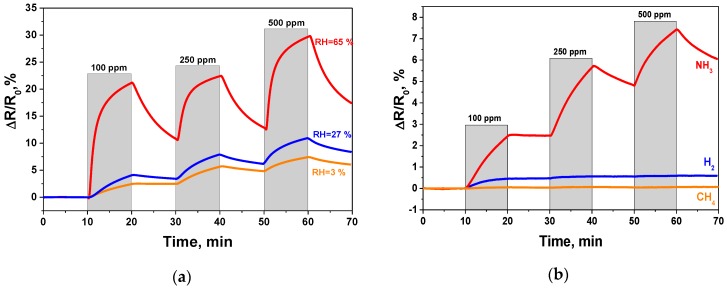
GO sensor response obtained at room temperature: (**a**) influence of increasing RH on response; (**b**) response towards different gases in dry air (RH = 3%).

One of the most important issues is the influence of RH on the sensor response. The increase of the RH leads to sensor response growth, which indicates the effect of the response increase after GO surface humidification. The response to 100 ppm, 250 ppm and 500 ppm of ammonia at 65% RH was equal to 22.2%, 22.5%, 29.6%, respectively. The response increase with RH can be caused by the improved ammonia adsorption on the GO surface in wet air. There are two types of ammonia adsorption on the GO sensor surface in dry and wet air. In [6,35] the authors suggested that in dry air, NH_3_ interacts with carboxylic and sulfonic groups with the formation of ammonium salts (NH_4_^+^). Alternately, in wet air, the chemical interaction of ammonia with functional groups is less active, and therefore the dissolution of NH_3_ as physical adsorption mainly takes place. This fact also confirms the stronger recovery of the GO sensor in wet air. The chemically adsorbed NH_3_ is hard to remove from the surface due to its stronger interaction with the functional groups, and the domination of physical ammonia adsoption makes recovery more effective. The measurements with RHs of 3%, 27% and 65% were carried out (see Figure 5a).

The comparison of the sensor response of different active materials for NH_3_ detection at various RH values and the data obtained in this work are presented in Table 1. The recovery of the sensor response under 100 ppm of NH_3_ in air with 3%, 27% and 65% of RH (during 10 min) was 2%, 14% and 49%, respectively. It is worth noting that the contact with ammonia either in wet air or in dry air does not change the sensing mechanism of GO, which is based on a hole depletion mechanism [19,36,37], where GO can be treated as a p-type semiconductor and the absorption of an electron-donating compound such as NH_3_ leads to the increase of sensor resistance. The desorption of chemically adsorbed ammonia which will make the sensor reusable can be performed using thermal heating [38], infrared light irradiation [39], etc.

Cross-sensitivity measurements were also carried out for the prepared GO sensor. The cross-sensitivity to methane and hydrogen was investigated. The results we obtained showed that the sensor possessed a higher response towards ammonia than to CH_4_ and H_2_ (see Figure 5b). The response curve of the GO sample to CH_4_ and H_2_ indicated the weaker adsorption of these gases on the GO surface in a comparison with NH_3_. This fact can be explained by the chemical nature of these compounds, where ammonia donates more electrons compared with hydrogen and methane. Since the sensor shows good selectivity towards ammonia, it can be possibly used for ammonia detection in air, N_2_, inert gases and their mixtures which are widely used in industrial processes (NH_3_ adsorption in metallurgy, the petrochemical industry and the chemical industry). Also, we can suggest that GO can be used as an active material for environmental protection near plants for NH_3_ production and in refrigeration plants, in which ammonia is still used. One of the promising applications of this sensor is for health and safety [40].

## 4. Conclusions 

The obtained results show a high potential of graphite oxide applied as an active material for ammonia gas sensors operating at room temperature. The highest response of the prepared GO-based sensor to ammonia in dry air was 10% for 1000 ppm of NH_3_. The sensor response increased during the growth of the relative humidity due to the enhancement of ammonia adsorption in wet air and it reached the values of 22.2%, 22.5%, 29.6% for 100 ppm, 250 ppm and 500 ppm of NH_3_ in air, respectively, at 65% relative humidity. The selectivity tests showed a higher response towards NH_3_ in a comparison with CH_4_ and H_2_. The obtained data confirm the potential of graphite oxide application in a non-reduced state for the detection of ammonia in a gas phase, which makes it possible to create cheaper sensors based on GO in comparison with graphene-like materials. For example, the combination of a cheap sensing material (graphite oxide) with a highly scalable and reproducible technique of screen-printing creates a highly versatile platform in the field of sensor devices.

## Figures and Tables

**Table 1:** GO ammonia sensor performance in a comparison with literature data.

Active Material for NH_3_ Detection	NH_3_ Concentration (ppm)	Sensor Response (%)	RH (%)	Temperature (°C)	Reference
Graphite oxide	500	30	65	25	This work
Single-wall carbon nanotubes	62.5	3	56	25	[41]
Single-wall carbon nanotubes	100	6	80	25	[32]
Multi-wall carbon nanotubes	500	1.9	3	25	[42]
Fluorinated graphene	10,000	10.2	n/a	25	[20]
CVD graphene decorated Ag nanoparticles	500	12.5	80	25	[43]
		16	100	25	
Reduced graphene oxide decorate by TiO_2_ microspheres	30	3.2	89	20	[44]
	30	3.5	17.8	22	
